# Mimicking exercise: what matters most and where to next?

**DOI:** 10.1113/JP278761

**Published:** 2020-01-14

**Authors:** John A. Hawley, Michael J. Joyner, Daniel J. Green

**Affiliations:** ^1^ Exercise and Nutrition Research Group Mary MacKillop Institute for Health Research Australian Catholic University Melbourne Australia; ^2^ Department of Anesthesiology and Perioperative Medicine Mayo Clinic Rochester MN USA; ^3^ School of Human Sciences (Exercise and Sport Sciences) The University of Western Australia Perth Australia

**Keywords:** AMPK, cardiovascular physiology, exercise mimetics, exercise physiology, PGC‐1, skeletal muscle

## Abstract

The past decade has witnessed growing scientific and commercial interest in the identification of bioactive oral compounds that mimic or potentiate the effects of exercise, so‐called ‘exercise pills’ or ‘exercise mimetics.’ These compounds have, to date, typically targeted skeletal muscle in an attempt to stimulate some of the adaptations to exercise induced by endurance training. Accordingly, they fail to impart many of the broad health protecting effects of exercise that are seen in tissues and organs other than skeletal muscle. In the context that multiple integrative regulatory and often redundant pathways have evolved to detect and respond to human movement, here we consider the complex challenges of designing a pill that might mimic the extensive range of exercise benefits. In particular, we consider the limits of the current ‘myocentric’ paradigm given the wide‐ranging array of impacts that exercise exerts on atherosclerosis and the cardiovascular system. We discuss the validity and limitations of the concept that low dose cardiovascular polypills, already in large scale trials, may represent one form of cardiovascular exercise mimetic. Finally, given that some calls for an exercise pill stem from a response to the perceived failure of expert advice, evidence‐based guidelines and current public health approaches, we explore possible strategies that might address the global rise in inactivity. In the event that a broad spectrum exercise mimetic might ever be developed, we discuss some generic issues related to adoption and adherence of therapeutic interventions.

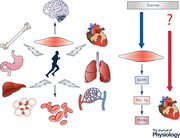

## Graphical abstract

**Abstract figure legend** Voluntary, dynamic exercise provokes widespread perturbations in multiple tissues and organs (left). In an attempt to mimic the effects of exercise training, compounds have focused on selected skeletal muscle signalling pathways (right), ignoring many of the health‐protecting effects of exercise in other organs and tissues, such as the heart.

## Introduction

Physical inactivity is the fourth leading cause of death globally, with almost one‐third of the world's population failing to meet the minimum prescription for health benefit (Hallal *et al*. 2012). The universal burden of physical inactivity is mounting, with 6–10% of all deaths from non‐communicable diseases worldwide attributable to physical inactivity, a figure that rises to 30% for conditions such as ischaemic heart disease (Lee *et al*. 2012). Recognizing the proven benefits of exercise on numerous health outcomes (Ruegsegger & Booth, 2018) and the trend towards increasing inactivity worldwide (Guthold *et al*. 2018), the past decade has witnessed growing scientific and commercial interest in the identification of bioactive oral compounds that mimic or potentiate the effects of exercise, so‐called ‘exercise pills’ or ‘exercise mimetics.’

The idea of taking a pill (or pills) to acquire the benefits of exercise in the absence of physical movement and energy expenditure has mass appeal for many sedentary individuals who, for a variety of reasons, do not obtain sufficient physical activity to improve their general health. It may also be attractive for ‘big pharma’ to view physical inactivity as a market to be medicalized for profit. But is an exercise pill a bioplausible, realistic or desirable goal? In this review we will discuss what is meant by an exercise pill/mimetic, with a view to expanding our understanding of the impact of human movement beyond its effects on skeletal muscle signalling pathways. We will consider the challenges of designing a pill that can mimic the impacts of exercise, given the multitude of signalling networks that have evolved to detect and respond to movement. Finally, given that some calls for an exercise pill reflect a genuine *cri de coeur* response to the failure of expert advice, evidence‐based guidelines and current public health approaches, we consider what might work to reverse our contemporary devolutionary indolence.

## What do we mean when we talk about exercise mimetics?

Considerable debate has centred around the nomenclature of what constitutes an ‘exercise pill’ or an ‘exercise mimetic,’ and a number of opinion pieces have weighed in on this topic (Goodyear, 2008; Richter *et al*. 2008; Warden & Fuchs, 2008; Booth & Laye, 2009; Carey & Kingwell, 2009; Church & Blair, 2009; Hawley & Holloszy, 2009; Fan *et al*. 2013; Craig *et al*. 2015; Wall *et al*. 2016; Fan & Evans, 2017; Guerrieri *et al*. 2017; Li & Laher, 2017; Weihrauch & Handschin, 2018). In the interests of space, we have summarized the main issues discussed in these commentaries with a view to informing a broader future dialogue.

First, and fundamentally, the use of the term ‘exercise pill’ or ‘exercise mimetic’ is a misnomer, inaccurate and misleading (Booth & Laye, 2009; Booth & Hawley, 2015). Exercise training provokes widespread perturbations in numerous cells, tissues and organs, conferring multiple health‐promoting benefits, and it is the multiplicity and complexity of these responses and adaptations that make it highly improbable that any single pharmacological approach could ever mimic such wide‐ranging effects (Hawley *et al*. 2014). The term ‘exercise mimetic’ should arguably be used to refer to interventions that simulate the broad array of adaptations and health benefits of exercise, recognizing the complex integrative physiological responses throughout the body that occur in response to exercise (Booth & Laye, 2009). The simplistic use of the term ‘exercise mimetic’ in this context creates unrealistic, if highly marketable, exaggeration of putative benefit. Current pharmacological compounds that purport to be ‘exercise mimetics’ (Narkar *et al*. 2008; Guerrieri *et al*. 2017; Choi *et al*. 2018; Amoasii *et al*. 2019) activate only a limited number of metabolic networks within skeletal muscle, without addressing impacts on atherosclerosis, the cardiovascular system or indeed other organ systems impacted by exercise. As such, terminology to describe the effect of any oral compound that purports to have ‘exercise‐like’ properties should be focused on the narrow range of biological pathway(s) activated by any given agent, so that the specificity and limited response to that drug are accurately defined (Booth & Laye, 2009; Carey & Kingwell, 2009).

A second issue arising directly from the imprecise nomenclature surrounding exercise pills/mimetics is the generic use of these terms, with no appreciation or insight given to distinct biological adaptations that occur in response to different modes of exercise (e.g. endurance‐ *versus* strength‐based, or concurrent training) and their divergent effects (i.e. increased oxidative capacity and fatigue resistance, muscle hypertrophy and strength). As such, the general public could be excused for thinking that exercise mimetics are a ‘one pill fits all’ and that the benefits of all types of exercise can be obtained from a single compound. This is a spurious notion that ignores the complexity and variability of both human adaptation (e.g. high *versus* low responders), and also the science underpinning exercise prescription for optimization of benefit. Whilst there has been an almost exclusive focus on discovering molecules that activate skeletal muscle pathways promoting an ‘oxidative/endurance phenotype’, little scientific enquiry has been devoted to compounds that could stimulate networks that preserve other aspects of systemic health, such as cardiovascular function (discussed subsequently). Realistically, what single pill could simultaneously increase skeletal muscle oxidative capacity and endurance, improve muscle strength and function, stimulate cardiovascular remodelling and enhance autonomic nervous system function?

## The problem of reductionism in the context of exercise mimetics

One thing we have learnt from the results of studies utilizing knockout animals is that no single ‘exercise gene’ or pathway exists (Booth & Laye, 2009) and that redundancy is a characteristic of complex human integrative systems (Joyner, 2011; Hawley *et al*. 2014). For example, the discovery of the inducible coactivator, the peroxisome proliferator‐activated receptor‐γ coactivator (PGC‐1α) in 1999 heralded a major breakthrough in unravelling the cellular events that promote mitochondriogenesis (Wu *et al*. 1999). Subsequent observations that many mitochondrial genes were reduced in PGC‐1α knockout mice, and that muscle‐specific overexpression of PGC‐1α activated genetic programmes characteristic of slow‐twitch muscle fibres (Lin *et al*. 2002), and improved endurance running capacity (Calvo *et al*. 2008), led to the assumption that PGC‐1α was the ‘master regulator’ of mitochondrial biogenesis (Wu *et al*. 2002). However, later work revealed that exercise‐trained PGC‐1α knockout mice still had all or many of the normal endurance‐based exercise adaptations (Leick *et al*. 2008; Rowe *et al*. 2012), suggesting that PGC‐1α was not mandatory for exercise training‐induced increases in skeletal muscle mitochondrial proteins. Of course, the fact that PGC‐1α is not necessary does not exclude that targeting the pathway might be a good thing.

**Figure 1:**
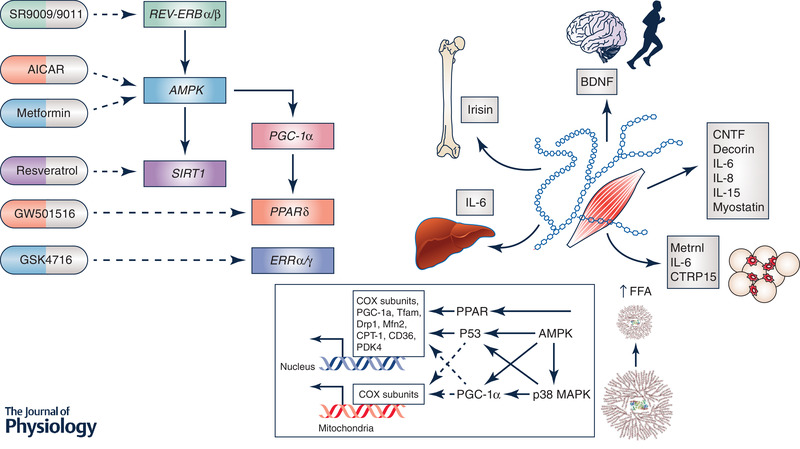
Multiple tissues and organ systems are affected by exercise, initiating diverse homeostatic responses The majority of exercise mimetics have targeted metabolic networks within skeletal muscle (left panel). The discovery of muscle ‘cross‐talk’ with multiple organs including adipose tissue, liver, pancreas, bone and the brain (right panel), has provided exercise biologists with a mechanistic framework for understanding how exercise mediates many of its beneficial whole‐body effects on health. While some myokines exert their actions on other organs in an endocrine fashion, others operate locally on skeletal muscle and thereby provide a feedback loop for the muscle to regulate its own growth and regeneration for adaptation to exercise training. In addition, muscle energy status (i.e. glycogen) exerts profound effects on acute regulatory processes underlying gene expression and cell signalling. As such, nutrient–exercise interactions have the potential to upregulate many biochemical pathways with putative roles in training adaptation. It is highly unlikely that any exercise pill/mimetic or combinations thereof could ever replicate the widespread effects of exercise on other organs/tissues, or respond to changes in the prevailing muscle fuel stores.

The multiple and often redundant responses associated with exercise (Hawley *et al*. 2014), the diverse signalling kinases that respond to these stimuli (Hoffman *et al*. 2015), the numerous downstream pathways and targets (Egan *et al*. 2016), and the complex spatial and temporal interactions between the various elements that combine to produce the integrated response to an exercise challenge (Bassel‐Duby & Olson, 2006) make it highly improbable that there would ever exist a single putative regulator driving any exercise phenotype. Indeed, a common feature of the so‐called ‘master regulators’ of metabolism (e.g. PGC‐1α) is that they are highly versatile, interacting with many different transcription factors to activate distinct biological programmes in different tissues (Lin *et al*. 2005). It is this very versatility that make these biological nodes of regulation a double‐edged sword, with their ultimate efficacy as therapeutic targets dependent on the ability to achieve biological and tissue specificity (Puigserver & Spiegelman, 2003). The recent discovery that contracting muscle releases myokines (Fig. 1) and is involved in ‘cross‐talk’ with other organs/tissues (adipose tissue releases adipokines in a similar fashion) also adds a level of complexity to any discussion of drugs/compounds to mimick or potentiate the effects of exercise (Eckel, 2019). Indeed, secretomic studies of muscle cells have shown the presence of hundreds of myokines (Hartwig *et al*. 2014), most of which have unknown functions. In addition to myokines, metabolites released by contracting skeletal muscle play an important role as ‘exercise factors’ that fine‐tune metabolic control. Such multiplicity, in our opinion, makes it highly unlikely that such metabolic cross‐talk could be evoked by artificial compounds.

A third issue is that maintaining a drug‐induced ‘metabolic overdrive’ for sustained periods could have potentially deleterious health effects (Weihrauch & Handschin, 2018), as constant activation of ‘exercise pathways’ can induce a chronic *catabolic* state due to the inhibition of protein synthesis and activation of autophagy (e.g. activation of AMPK and the inhibition of the mammalian target of rapamycin, mTOR, a key regulator of protein turnover). The foundations for exercise‐induced adaptation are episodic periods of metabolic overload, followed by adequate rest/recovery for restoration of muscle substrates, muscle repair and the regeneration necessary for any adaptation to occur. We are not aware of any evidence that pharmacological compounds could mimic such effects, although it may be theoretically possible to do so. The variability observed between humans in response to any drug, along with consideration of the optimal dose or doses required to maintain activation/repression of targeted biochemical pathways, make long‐term prescription of an exercise pill/mimetic problematic and challenging, just as it does for existing drug approaches. This scenario is exacerbated if several ‘mimetics’ need to be taken simultaneously. As suggested by Spiegelman (2018), perhaps specific pathways induced by exercise can be dosed well beyond what is naturally produced during exercise, a challenge taken up by the NIH through the recent launching of its programme ‘Molecular Transducers of Physical Activity.’

## What do we want from an exercise mimetic? Why is exercise worth mimicking?

**Figure 2:**
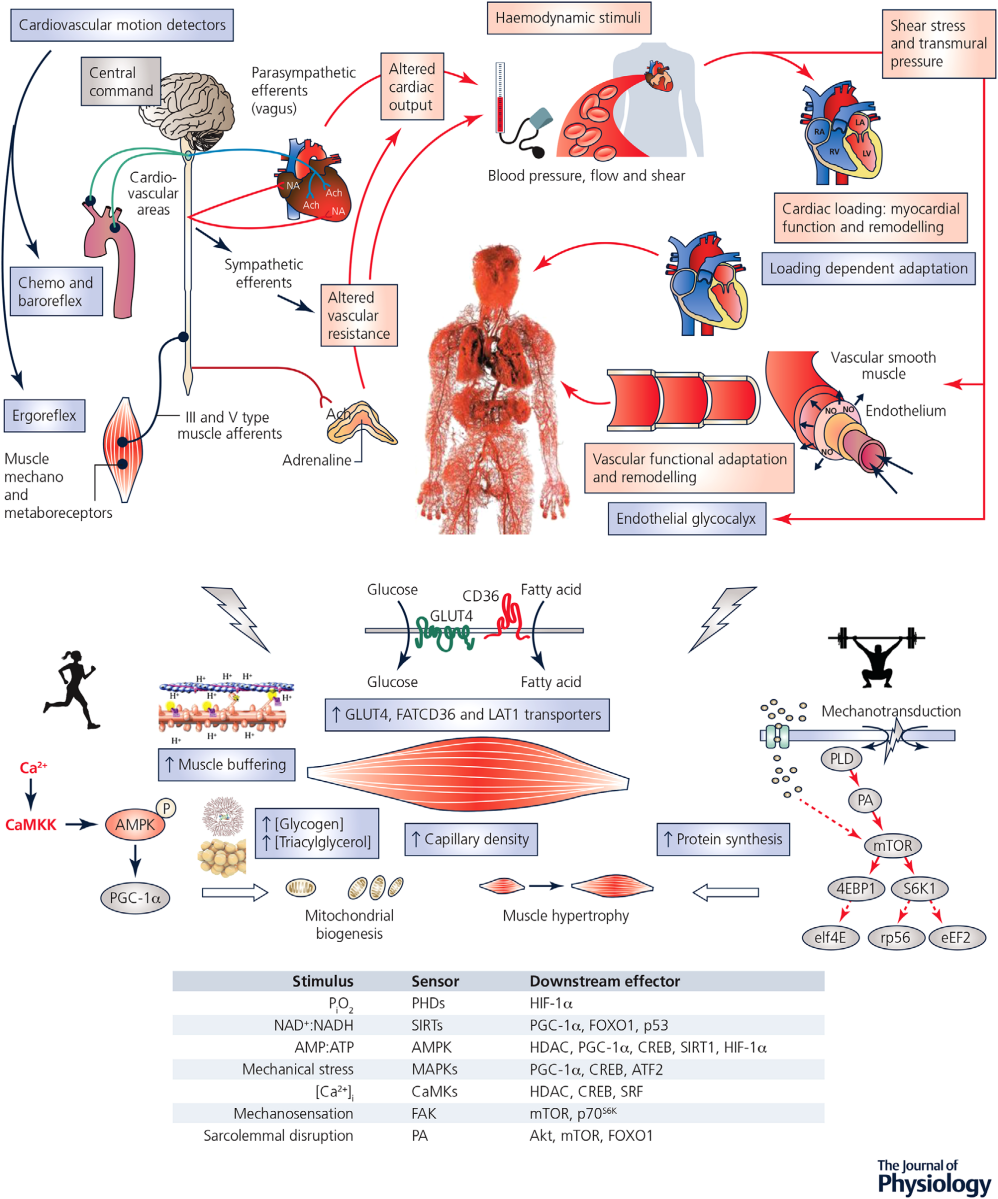
Central (cardiovascular) and peripheral (skeletal muscle) adaptations to exercise training Cardiovascular adjustments to exercise (upper panel) require an intact autonomic nervous system and are driven by three major signals: (1) feedforward ‘central command’ related to motor output, which activates selected areas in the brainstem cardiovascular (and respiratory) centres to stimulate increases in heart rate, blood pressure and ventilation; (2) afferent feedback from thinly myelinated and unmyelinated type III and IV afferents in contracting muscles that increase sympathetic activation; and (3) baroreceptors in the carotid sinus and aortic arch that provide feedback on blood pressure to the brainstem cardiovascular centres. A common feature of the cardiovascular responses to exercise is the detection of motion‐related signals, such as the movement of blood (shear stress) and pressure in the heart and arteries (transmural pressure). Such haemodynamic signals transduce acute and chronic adaptation. Redundancy and compensatory regulation are key characteristics of these biological systems which act to preserve physiological homeostasis. Contraction‐induced adaptations to exercise training, and modulators of gene expression in skeletal muscle (lower panel), that ultimately lead to functional improvements in exercise capacity/performance and drive alterations in phenotype.

It is puzzling that the focus of exercise mimicry has been on promoting mitochondrial biogenesis in skeletal muscle, when there is a much larger and more compelling landscape for the benefits of exercise in humans (see Fig. 2). The global causes of disease study reported around half of the top 15 causes of death worldwide possess an atherosclerotic aetiology, including ischaemic heart disease and cerebrovascular disease, dementia, diabetes, kidney disease and hypertension (GBD 2015 Mortality and Causes of Death Collaborators, 2016). In all cases, the age‐related incidence of these diseases decreased in the decade between 2005 and 2015, yet global impact *increased* due to changes in population ageing and growth. Additionally, there is at least some emerging evidence that age‐specific rates of atherosclerotic disease are once again on the rise in countries like the United States that have had high levels of obesity for many years (Khan *et al*. 2018). Whilst not dismissing the impacts of exercise on skeletal muscle, perhaps a more compelling question is ‘to what extent might an exercise mimetic benefit the cardiovascular system?’ Specifically, since each of these end‐organ manifestations is largely related to a common disease process, what effects could an exercise mimetic have on the most common, costly and deadly global disease in the modern world, namely atherosclerosis?

Atherosclerosis is a disease that begins in the first decade of life (Enos *et al*. 1955; Stary, 1989; Tuzcu *et al*. 2001), with important genetic and intra‐uterine pre‐determinants. It is detected extremely late and contemporary management largely treats it as a problem limited to isolated organs or focal lesions (Kannel *et al*. 1998; Fox *et al*. 2004; Smolina *et al*. 2012). It is a systemic disease (Stone *et al*. 2011), so exercise is an appealing therapy because it has systemic effects (Green *et al*. 2002), and it can be applied in an early prevention context (Watts *et al*. 2004). Importantly, exercise has both direct and indirect benefits in terms of the health of the heart and the artery walls (Green *et al*. 2008).

Early literature on the cardiovascular benefits of exercise focused on the effects of exercise on cardiovascular risk factors; that is, artery health was perceived as being a consequence of the impact that modification of risk factors exerted in the vessel wall. In general, these studies found that exercise had beneficial effects on individual risk factors and that the magnitude of benefit depended upon the nature of the patients (e.g. raised risk factors at entry or relatively healthy, young or old, male or female) and the nature of the intervention (endurance or resistance training, high or low intensity, duration, frequency, etc.). A general conclusion from this vast body of research is that, although, on average, exercise has beneficial effects on reducing risk, the magnitude of exercise‐induced benefit is modest compared to the gains achieved from pharmacological therapy (e.g. statins, angiotensin converting enzyme inhibitors [ACEI], angiotensin receptor blockers [ARB]) (Thompson *et al*. 2003). This is not to suggest that exercise is an inferior intervention, especially since it can be considered a ‘polypill’ capable of modifying multiple risk factors at once (Joyner & Green, 2009). However, for each individual risk factor, with the possible exception of exercise *versus* metformin for diabetes prevention (though not treatment), the drugs typically ‘win’. Why not simply treat each risk factor using the blockbuster drug? Or better yet, develop a drug combination (pharma polypill) to get the job done?

## If exercise is a cardiovascular polypill, is a pharmacological polypill a better strategy?

If we broaden the concept of an exercise mimetic beyond skeletal muscle mitochondrial adaptations to include its impacts on the cardiovascular system, perhaps we already have a form of exercise mimetic? The concept that a polypill containing low doses of several compounds might afford broad‐based protection was first proposed in the early 2000s (Wald & Law, 2003). Such a polypill would include compounds that lower blood lipids, reduce blood pressure, are anti‐thrombotic, blunt autonomic responses, and lower blood glucose concentration. The argument for such a polypill is that in large randomized clinical trials, low doses of many drugs in these classes have minimal side effects, but when administered together they would act synergistically to reduce mortality from cardiovascular disease. Such a polypill would also activate many of the protective factors and mechanisms (both traditional and non‐traditional) engaged by exercise (Fiuza‐Luces *et al*. 2013), with skeletal muscle mitochondrial biogenesis being an obvious exception to this assertion (see below).

Along, these lines, the original polypill modelling by Wald and Law (2003) asserted that ‘The Polypill strategy could largely prevent heart attacks and stroke if taken by everyone aged 55 and older and everyone with existing cardiovascular disease. It would be acceptably safe and with widespread use would have a greater impact on the prevention of disease in the Western world than any other single intervention.’ Recent clinical trials have shown that the magnitude of risk factor reduction evoked by short term use of a polypill (∼80%) is similar to those predicted in the original modelling paper (Wald *et al*. 2012). Retrospective analyses on a suite of polypill compounds suggest improved long‐term survival after acute coronary syndrome or myocardial infarction (Oliveras Vila *et al*. 2014). Parenthetically, individuals in Sweden who have persistently advantageous risk factor profiles across a 20 year period (low blood pressure, physically active, appropriate diet, non‐smokers) exhibited an 80% lower risk of myocardial infarction than those who possess none of these virtues (Akesson *et al*. 2014). A similar analysis in the USA revealed a >12 year longevity benefit beyond the age of 50 years for those in the virtuous grouping (Li *et al*. 2018). A recent primary and secondary prevention trial of a low dose polypill in 6838 subjects aged 40–75 years found a 40% reduction in the risk of major cardiovascular events in individuals without a prior history of cardiovascular disease. Adherence based on blister pack count was generally excellent (80%) and adverse events were low. In a subgroup with even higher adherence, the reduction in events was close to 60%. These findings may, if anything, be an underestimate of benefit, since the comparator group received a healthy lifestyle education intervention (Roshandel *et al*. 2019).

These observations are consistent with the potential for a marked decrease in mortality with broad based use of the cardiovascular polypill strategy. If the goal of an exercise mimetic is to improve cardiometabolic risk among individuals who cannot or will not exercise, then a polypill approach may be considered a viable and well tolerated alternative. Additionally, it is clearly possible to formulate such a polypill with already approved and extremely low‐cost components designed for once per day dosing.

## Can a polypill that targets risk factors *fully* mimic the cardiovascular benefits of exercise?

An unanswered question is whether the addition of exercise to a low‐dose polypill that emulates exercise‐like effects on cardiovascular risk factors can provide additive benefit. We speculate that this may indeed be the case, given the existence of a ‘risk factor gap’. In the mid‐2000s we pointed out that the beneficial effects of physical activity, in terms of its impact on all‐cause and cardiovascular mortality, could not be fully accounted for by considering the effects on cardiovascular risk factors (Green *et al*. 2008; Joyner & Green, 2009). Whilst it is generally accepted that humans who have high levels of either occupational or recreational physical activity have a 30–40% reduction in both all‐cause and cardiovascular mortality (Morris *et al*. 1953; Blair & Morris, 2009; Moore *et al*. 2012), physical activity and/or exercise training have relatively modest effects on traditional risk factors (lipids, blood pressure, glucose concentrations) associated with cardiovascular disease (Thompson *et al*. 2003). The cumulative effect of physical activity and/or exercise on these risk factors explains only 30–50% of the reductions in all‐cause and cardiovascular mortality seen as a result of physical activity (Mora *et al*. 2007; Hamer & Stamatakis, 2009).

Because a substantial fraction of the protective effects of exercise are unaccounted for by modification in traditional risk factors, other mechanisms must be invoked to explain the unexplained positive effects of exercise on mortality. The possible additional effects of exercise on key protective mechanisms include: (1) improved endothelial function which is anti‐atherogenic, pro‐vasodilatation, anti‐inflammatory and anti‐thrombotic; (2) remodelling and increased size of large blood vessels like the coronary arteries along with increased collateral circulation; (3) improved autonomic balance to the heart which likely has anti‐arrhythmic effects; and (4) psychological effects related to mood and autonomy. In this context, for an exercise mimetic to emulate the cardiovascular effects of exercise it would have to have broad based effects on cardiovascular risk factors, and also mimic exercise effects on other organ and regulatory systems.

## Can a polypill or exercise mimetic activate evolutionary pathways geared to detect movement?

The Nobel prize‐winning discoveries showing that endothelial cells produced paracrine hormones with myriad anti‐atherogenic effects introduced the important concept that exercise, by virtue of its impacts on the movement of the blood, has direct effects on artery health and atherosclerotic risk (Green *et al*. 2017). Endothelial cells detect shear stress forces that trigger their activation (Pohl *et al*. 1986; Berdeaux *et al*. 1994; Dawson *et al*. 2010; Gielen *et al*. 2010). If the shear stimulus is episodically repeated, then chronic adaptation occurs with upregulation of paracrine generating systems (Green *et al*. 2004). In this way, increases in blood *movement* is a trigger or stimulus to upregulation of endothelial function which, in turn, is anti‐atherogenic by virtue of impacts of platelet adhesion and activation, transmigration of monocytes to the subintimal space, decreased macrophage transformation, decreased smooth muscle cell proliferation, and transmigration and anti‐oxidant impacts which diminish low‐density lipoprotein oxidation and foam cell formation. Longer term shear stress‐mediated activation of the endothelium induces structural remodelling of the artery wall, favouring enhanced blood flow (Langille & O'Donnell, 1986; Green *et al*. 2017).

Although distinct forms of shear stress can induce distinct types of artery functional and structural adaptation (Thijssen *et al*. 2009; Newcomer *et al*. 2011), in general it is accepted that episodic increases in shear are highly beneficial in terms of arterial health. Whilst shear forces can be induced through contrived laboratory means such as repeated heating (and this has beneficial effects of artery function and health (Green *et al*. 2010; Naylor *et al*. 2011), the most common form, *indeed the natural and evolutionary stimulus that triggers arterial shear stress, is* human movement. This is an important point in the context of those who would propose an exercise mimetic. If humans have evolved detector mechanisms that are based on motion, such as the movement of blood across endothelial cell membranes, how does one mimic such pathways in a resting individual who is administered a pill? Indeed, endothelial activation and adaptation is but one example of a physiological system based on the detection of a biophysical stimulus. Arteries are also sensitive, and adapt to, changes in transmural wall pressure (Atkinson *et al*. 2015), whilst specialized cardiopulmonary and arterial baroreceptors monitor blood pressure and induce autonomic responses (baroreflexes). Ergoreceptors trigger cardiovascular responses in skeletal muscle, whilst the heart itself responds to changes in chamber loading conditions (volume and pressure) by modifying its function and remodelling (Spence *et al*. 2011).

Several truths emerge for those who enthusiastically promote the merits of an exercise pill on the basis of substances that solely mimic muscle‐centric effects. Exercise is not simply a stimulus for upregulating metabolic pathways in skeletal muscle, but, instead, has widespread effects on multiple organ systems including myriad impacts on the cardiovascular system. The arteries and the heart are end‐organs in terms of the impact of exercise; they do not simply benefit from skeletal muscle serfdom. Exercise is a stimulus that directly affects cardiovascular function and, by virtue of this, arterial and cardiac adaptation. This occurs indirectly because exercise modifies risk factors in ways that enhance endothelial and cardiac function, but also more directly because changes in systemic haemodynamics are directly sensed and responded to by cardiovascular structures, including those in the walls of the arteries and the heart.

## What evidence is there that humans would adhere to taking an exercise pill, or multiple pills?

The preceding discussion highlights the fact that, as a consequence of the blockbuster era of drug development that occurred from the 1980s to the 2000s, we have highly effective medications for the treatment of blood pressure (ACEI, ARB), cholesterol (statins) and clotting. Thanks to the pharma‐driven profit motive, the evidence‐based benefits of these medications are extremely well known. Yet there is clear evidence that adherence to these medications is very poor: among patients with chronic illness, ∼50% or more of patients do not take medications as prescribed (Brown & Bussell, 2011). This may partly be due to perceived or under‐appreciated side effects (e.g. myalgia for statins), the cost of lifelong commitment to prescription and/or the complexity of managing multi‐drug prescriptions. It is worth noting that, in a recent trial of a polypill combining three medications, adherence was excellent compared to that associated with multiple drug regimes (Roshandel *et al*. 2019). This point is germane for those advocating the development of an exercise pill, or pills, when these would need to be added to the existing multiple‐drug regimens common in patients with chronic diseases. In the context of adherence and tolerability, it is also worth noting that the vast majority of skeletal muscle mimetics to date have not been tested in humans, with knowledge of the effects of these compounds on biomarkers of health and exercise capacity based entirely on preclinical findings in rodents (Li & Laher, 2017). In several cases, severe side effects associated with some of the first‐generation ‘mimetics’ have precluded their further clinical development (Fan & Evans, 2017). Whether the outcomes observed in animal models (using reductionist ‘loss‐of‐function and/or gain‐of‐function’ approaches) translate into humans is a leap of faith.

In summary, increasing the effectiveness of adherence to existing evidence‐based and highly effective medications would, arguably, have a greater impact on the health of the population than the invention and addition of another pill, or multiple pills, to ‘cure’ inactivity. In the context that less than half of patients with chronic diseases currently adhere to their multi‐drug regimes, it seems a flawed strategy to add further drugs that mimic exercise; the addition of multiple ‘exercise mimetics’ necessary to mimic the broad spectrum of impacts of exercise in multiple organ systems is even more problematic.

Finally, the issues raised in this section reveal a logical sinkhole for those who would promote a pharmacological fix for behavioural problems. Ergo: *Let's fix a behavioural issue, inactivity, using a pill! But people are likely to be resistant to taking our new drug. Let's fix non‐adherence to taking pills, a behavioural issue, with a new pill …..?* What evidence do the proponents of the development of a pill to fix a behavioural problem, inactivity, have that people would behave well enough to adhere to it? Would these proponents also recommend the development of a drug that manages another behavioural issue, drug adherence, with a new drug aimed at making people compliant with advice to take their medications?

## Where to from here?

**Figure 3:**
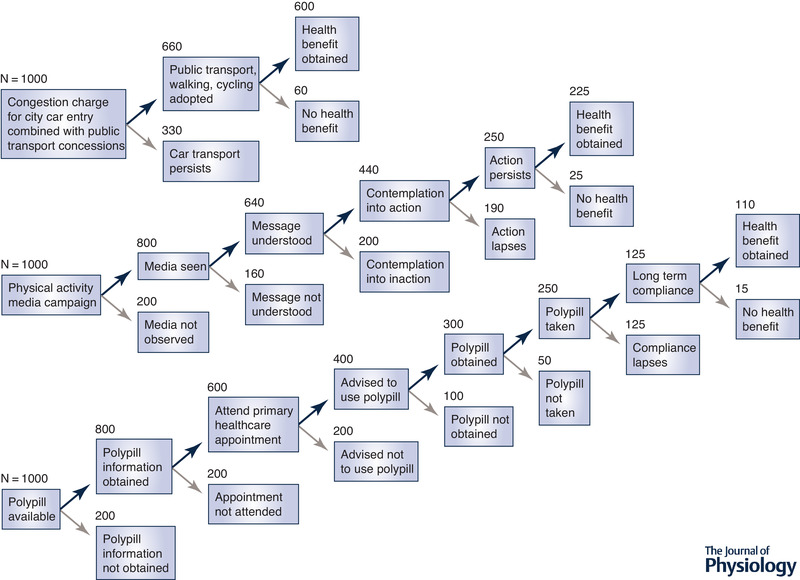
Comparison of the theoretical (and admittedly speculative) impact of low‐ (upper panel) and high‐ (middle panel) agency interventions to modify levels of physical activity (based on the paper of Adams *et al*. 2016) The impact of a polypill is also included for comparative purposes (lower panel). The upper panel demonstrates the impact of introducing a central city congestion charge, similar to that introduced in London in 2003. Figures are estimates based on the observation that two‐thirds of trips through the city are made by public transport, walking or cycling (https://theconversation.com/london-congestion-charge-what-worked-what-didnt-what-next-92478). The middle panel reflects the estimated impacts of public health media strategies to modify physical activity levels (a high‐agency public health intervention). The bottom panel illustrates the proposed pathway for adoption and beneficial impact of a polypill strategy. Numbers are for illustrative and comparative purposes and are speculative and not evidence‐based.

In this review we have discussed the biological and cultural limitations of the current skeletal muscle‐centric focus on exercise mimetics. Whilst able to mimic some of the health aspects of exercise, such mimetics present a narrow perspective in the fight against the inactivity epidemic and ignore the complex adaptive responses associated with many forms of exercise in multiple organ systems. At best, they might represent a partial exercise mimetic. From a cultural and behavioural perspective, a host of issues related to adoption and adherence that plague almost every therapeutic intervention are likely to be at play should an exercise pill ever be developed (see Fig. 3). In this context, a focus on so‐called low‐agency interventions that promote physical activity seems like an attractive strategy (Adams *et al*. 2016). The extent to which the political will exists to implement evidence‐based strategies remains to be demonstrated.

## Additional information

### Competing interests

The authors have no conflicts of interest to declare.

### Author contributions

All authors have approved the final version of the manuscript and agree to be accountable for all aspects of the work in ensuring that questions related to the accuracy or integrity of any part of the work are appropriately investigated and resolved. All persons designated as authors qualify for authorship, and all those who qualify for authorship are listed.

### Funding

None.
